# Lipid Multilayer Grating Arrays Integrated by Nanointaglio for Vapor Sensing by an Optical Nose

**DOI:** 10.3390/s150820863

**Published:** 2015-08-21

**Authors:** Troy W. Lowry, Plengchart Prommapan, Quinn Rainer, David Van Winkle, Steven Lenhert

**Affiliations:** 1 Department of Biological Science and Integrative Nanoscience Institute, Florida State University, 89 Chieftan Way, Tallahassee, FL 32304, USA; E-Mails: twl10@my.fsu.edu (T.W.L.); qr13@my.fsu.edu (Q.R.); 2 Department of Physics, Florida State University, 77 Chieftan Way, Tallahassee, FL 32304, USA; E-Mails: pp11d@my.fsu.edu (P.P.); rip@phy.fsu.edu (D.V.W.)

**Keywords:** lipid, lipid multilayer, sensor, nanointaglio, nanofabrication, AFM, sensor-array, materials integration, microcontract printing, nanotechnology

## Abstract

Lipid multilayer gratings are recently invented nanomechanical sensor elements that are capable of transducing molecular binding to fluid lipid multilayers into optical signals in a label free manner due to shape changes in the lipid nanostructures. Here, we show that nanointaglio is suitable for the integration of chemically different lipid multilayer gratings into a sensor array capable of distinguishing vapors by means of an optical nose. Sensor arrays composed of six different lipid formulations are integrated onto a surface and their optical response to three different vapors (water, ethanol and acetone) in air as well as pH under water is monitored as a function of time. Principal component analysis of the array response results in distinct clustering indicating the suitability of the arrays for distinguishing these analytes. Importantly, the nanointaglio process used here is capable of producing lipid gratings out of different materials with sufficiently uniform heights for the fabrication of an optical nose.

## 1. Introduction

Biological systems are uniquely capable of distinguishing a wide variety of different analytes in complex environments. Typically, this sensing is carried out at a cellular level by detection of a binding event to a cellular membrane by means of a receptor, which subsequently triggers a signal amplification cascade within the cell leading to a cellular response. Lipid multilayer gratings are a promising approach to the recreation of biological sensing capabilities in synthetic sensors. In this case, diffraction gratings are formed from biological lipids, and the binding of analytes to these gratings results in a shape change in the lipid multilayer which can be read out optically by monitoring the intensity of light diffracted from the grating. We have previously shown that lipid multilayer gratings are sensitive to vapors and that biotinylated gratings are capable of detecting the binding of the protein streptavidin at concentrations down to 500 picomolar [1].

In order to use lipid multilayer gratings for multiplexed sensing, multiple different materials must be integrated onto the same surface with reliably controlled heights. The control of lipid multilayer height [2] is particularly important as grating height has been observed to affect the sensor response [1]. We first used lipid dip-pen nanolithography [3,4] for lipid multilayer grating fabrication. That method is ideal for prototype development because arbitrary patterns can be drawn, and lipid multilayer heights can be varied in a single experiment. However, we have been unable to produce lipid grating arrays out of multiple different lipids with uniform heights using dip-pen nanolithography because different lipids and tips require different patterning conditions. Other methods of lipid multilayer pattering include dewetting on a prepatterned substrate [5], hydrogel stamping [6,7] evaporative edge lithography (EEL) [8] and nanointaglio [9,10].

**Figure 1:**
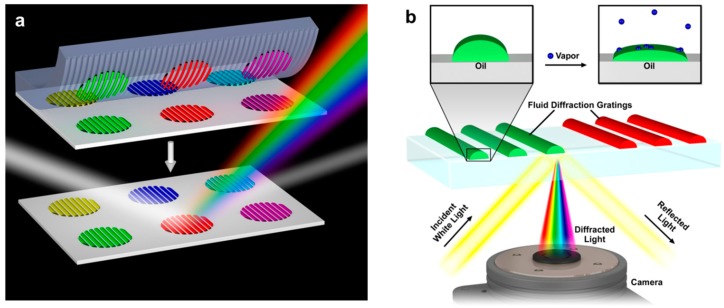
Nanointaglio printing of lipid multilayer grating arrays and their use as an optical nose for vapor sensing: (**a**) Schematic illustration of the use of nanintaglio to pattern six different lipid multilayer gratings onto polystyrene; (**b**) Schematic of the sensing process: exposure to vapor causes a shape change in the chemically distinct lipid grating elements (labeled “oil”).

Nanointaglio is a recently developed process that is suitable for fabrication of lipid multilayer gratings out of multiple materials with uniform heights. It is based on carrying out microcontact printing in an intaglio mode where the ink is transferred from the recesses of the stamp rather than from the protrusions, the latter being a relief printing mode that is commonly used for monolayer patterning [11]. We have recently shown that three different fluorescently labeled inks can be integrated onto a nanointaglio stamp using a robotic pin spotter originally designed for printing DNA microarrays [10,12]. These different inks can then be simultaneously printed resulting in patterns with uniform thickness. Here we extend the nanointaglio process for the printing of six chemically distinct lipid mixtures with suitable uniformity for an optical nose [13,14,15,16] measurement to distinguish three different vapors in air and pH under water, as illustrated in Figure 1. While the sensor chip is not reusable if the pattern gets destroyed, the fabrication of the sensor chip is cost-effective. A batch of about 50 chips can be produced in the laboratory at a material cost of less than 5 US dollars.

## 2. Experimental Section

### 2.1. Lipid Ink Preparation

Lipids used for arraying into lipid multilayer grating patterns were 1,2-dioeoyl-*sn*-glycero-3-phosphocholine (DOPC); 1,2-dioleoyl-3-trimethylammonium-propane (chloride salt) (DOTAP; 1,2-di-(9Z-octadecenoyl)-*sn*-glycero-3-phospho-L-serine (sodium salt) (DOPS); 1,2-dioleoyl-*sn*-glycero-3-phosphoethanolamine-N-lissamine rhodamine B sulfonyl (DOPE-RB). These were purchased from Avanti Polar Lipids, Inc. (Alabaster, AL, USA) Chloroform solutions of the different lipids were mixed to obtain the desired molar ratios. Six different lipid ink formulations were prepared by mixing the lipids at different molar ratios. When making the liposomal formulations, chloroform was evaporated off under a nitrogen stream, then further dried in vacuum overnight to form a thin film of lipid on the bottom of the glass vials. After drying, water was added to the vials containing the dried lipids. Samples were then lightly vortexed for 10 s, then sonicated for 10 min. After sonication, vortexing was used as needed to ensure suspension.

### 2.2. Substrate Preparation

Sterile polystyrene petri dishes were used as received directly out of the packaging (VWR, Radnor, PA, USA). PMMA sheets (Notz Plastics, Inc., Bienne, Switzerland) Substrates used for AFM of DOPC gratings were prepared by sonicating for ten minutes in isopropanol and then water followed by drying under a steady stream of nitrogen.

### 2.3. Microarraying

The different lipid solutions were microarrayed from standard 384 well microtitre plates (Axygen, Inc., PMI110-07 V1., Union city, CA, USA) using a BioRobotics pinspotter model BG600 (BioRobotics Ltd., Comberton, Cambridge, England) onto polystyrene, using a 200 µm 4 × 4 stainless steel microspot pin tool. After printing each lipid formulation, wash steps of 60 s in acetone and 60 s in water, followed by 20 s of drying ensured no cross contamination.

### 2.4. Characterization and Imaging Techniques

Diffraction and fluorescence images of patterned lipid gratings were captured using a Ti-E epifluorescence inverted microscope (Nikon Instruments, Melville, NY, USA) fitted with a Retiga SRV (QImaging, Surrey, BC, Canada) CCD camera (1.4 MP, Peltier cooled to −45 °C). For imaging the diffracted light, a fiber-optic white light source (Eco Light 150, MK Photonics, Albuquerque, NM, USA) was positioned to illuminate the sample at an angle of about 50° from perpendicular and images were captured in brightfield microscopy. Heights and topography of the lipid prints were measured using tapping mode with a Dimension Icon AFM (Bruker, Billerica, MA, USA) and tapping mode AFM cantilevers (FESPA, 8 nm nominal tip radius, 10–15 μm tip height, 2.8 N/m spring constant, Bruker, Billerica, MA, USA).

### 2.5. Data Analysis and Vapor/pH Sensing

The time dependency of the diffracted light intensity was recorded at one frame per two-second interval. These videos (e.g., Supplementary video 1) revealed that some pixel intensities varied non-monotonically. These pixels were determined to occur in regions where fluorescence in the red channel taken before analyte exposure was higher than average and the diffracted intensity appeared spatially non-uniform, which indicative of a larger multilayer height [2]. Therefore, pixels with fluorescence intensity higher than average were eliminated from the diffraction data analysis by assigned an intensity of zero in the green diffraction channel. For principal component analysis (PCA) [17] relative diffraction intensities were extracted using ImageJ (downloaded from the NIH website, http://imagej.nih.gov/ij/) from each image record for times of 20 and 40 s from exposure to analyte. This created a fingerprint of 12 total parameters (two times for each of six samples) for PCA. PCA was carried out using OriginPro^®^ 9, consisting of the 12 parameters and five trials for each solvent (15 total experiments). To analyze changes in pH, the experiment was repeated 6 times. All 6 of the pH experiments have an initial 10 min run for pH 5.8. Three of the experiments were used as PCA data for pH 5.8, trials 1 and 2, which were then used for pH 12.0 and trial 4 that was used for pH 2.0. Notice that there are three PCA points for each pH, this means that for 3 experiments the pH was adjusted initially at 5.8 to 2.0 and 3 where pH was adjusted initially from 5.8 to 12.0 after 10 min. For each pH (2.0, 5.8 and 12.0), relative diffraction intensities were extracted for each image at times 100 s and 350 s. PCA values were then extracted as described above. While more experiments would enable better PCA mapping, it is not without precedent that three to five repetitions is extensive enough for newly established sensor array devices [13,14,18]. Scree plots from each PCA map were used to quantify the variance for the principal component analysis. Two principal components account for 96.36% of the variance in the solvent experiments and 86.46% of the variance in the pH experiments. Furthermore, well separated clustering in principal component space is observed.

## 3. Results and Discussion

### 3.1. Lipid Multilayer Fabrication

**Figure 2:**
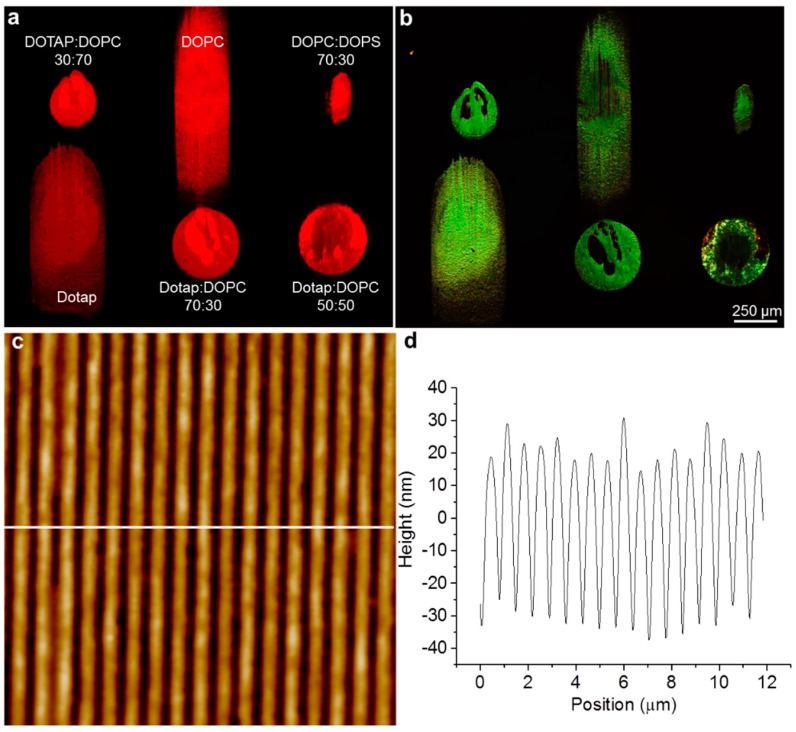
Lipid multilayer sensor array fabrication: (**a**,**b**) Fluorescence and green diffraction images, respectively of a patterned lipid multilayer grating sensor array onto poly(methyl methacrylate) (PMMA) sheets. Each lipid formulation is able to be printed using nanointaglio, despite the difference in charge and fluidity of each formulation; (**c**) AFM micrograph of a small section of the Dotap:DOPC 30:70 gratings shown in Figure 2a,b Gratings were patterned with a period of 700 nm and demonstrate good quality control for the stamped lipid material; (**d**) Section analysis of Figure 2c shows each of the gratings to be around 50 nm tall.

Multiple chemically-different lipid multilayer grating elements were integrated onto a surface by nanointaglio. Figure 2a,b show fluorescence and diffraction micrographs, respectively, of the integrated lipid multilayer grating sensor chip. Nanointaglio allows many different lipid inks to be printed onto the same chip, despite the dissimilarities in charge and fluidity of the different lipid formulations. Figure 2c,d show an AFM micrograph and corresponding sectional analysis of a small region of the Dotap:Dopc 30:70 lipid grating shown in Figure 2a,b. Using nanointaglio, high precision pattern control is possible with lateral features near the wavelength of light for high diffraction efficiency and nanometer control of the lipid grating heights. Control of lipid grating topography is determined by the stamp geometry. Furthermore, ink is transferred from the recesses of the grooves rather than the relief structures (like traditional microcontact printing), therefore, the intaglio stamp deposits lipid inks patterned with greater thickness (~10×) than that of a traditional lipid bilayer.

### 3.2. Sensing of Organic Vapors and Humidity

We demonstrate the use of nanointaglio to integrate lipid multilayer gratings composed of multiple different lipids to carry out an artificial-nose type of detection. The grating arrays were printed onto a substrate using nanointaglio (Figure 2a,b). Large uniform regions can be demonstrated by the strong diffraction intensity (Figure 2b) while local variations in grating features or non-uniform stamping areas are shown as having minimal to no diffraction intensity. An array of 6 different lipid formulations (arrayed as specified in the figure) was used to detect different common volatile organic compounds (VOCs) and humidity in saturated atmospheres by monitoring the diffraction intensity.

**Figure 3:**
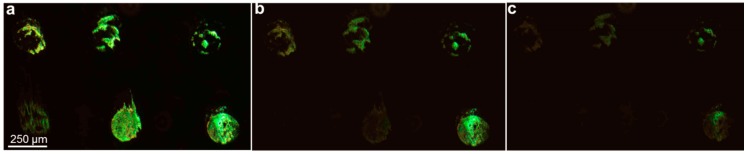
Sensor Response. (**a**) Initial diffraction intensity of six different lipid formulations printed onto polystyrene. The sensor array is composed of the same lipid formulations as in Figure 2a; (**b**) Upon exposure to ethanol vapor for 20 s; (**c**) 40 s, each lipid uniquely interacts with the solvent, causing different proportions of diffraction intensity loss, depending upon the lipid composition.

The sensor response for lipid grating-vapor reactivity is shown in the green diffraction micrographs in Figure 3. Figure 3a–c demonstrate a time lapse of initial exposure to vapor, 20 s after exposure and 40 s after exposure, respectively. Specific lipid compositions behave differently from both solvent to solvent and from composition to composition. S.Video. 1 shows the entire sensor response.

Detection of the changes in lipid multilayer structure induced by vapor exposure was observed by diffraction intensity as a function of exposure time. Diffraction readout is an appropriate readout since even small changes in grating height provide pronounced changes in diffraction intensity [1]. While the general overall trend of lipid grating intensity decreases over time, some pixels behaved anomalously, exhibiting non-monotonic variations. These anomalous pixels are thought to be caused by light scattering during changes in the lipid surface tension caused by dewetting and solution inhomogeneity, if applicable. To decrease noise in the results and improve the reproducibility of vapor patterns in principal component space, these pixels have been excluded from the grating response signal. The procedure used is described in the data analysis section.

### 3.3. Principal Component Analysis Demonstrates Unique Clustering of Different Vapor Exposure

**Figure 4:**
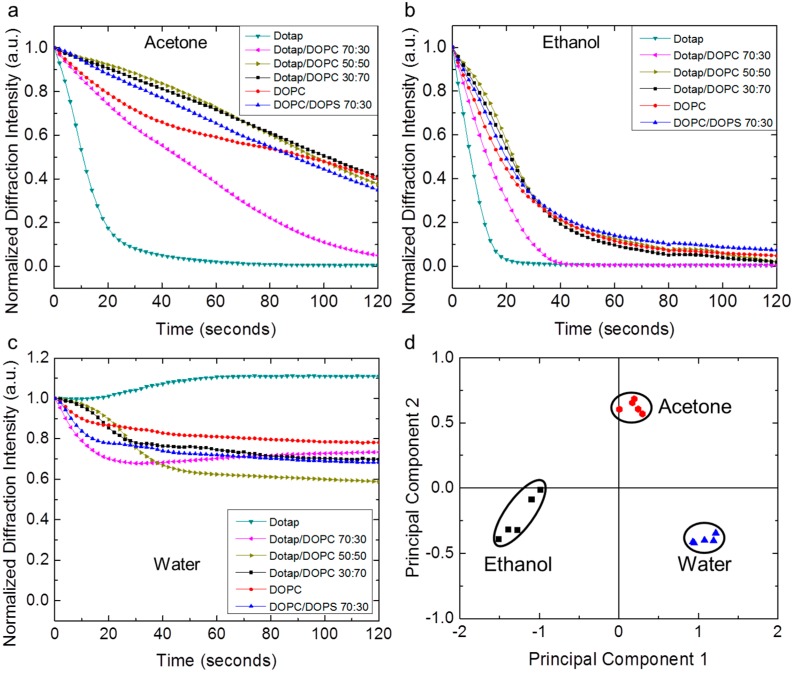
Principal Component Analysis produces separated clusters of lipid grating responses to different volatile organic compounds (VOCs) and humidity. (**a**–**c**) Individual responses to acetone, ethanol and water vapors, respectively for the six sensor elements composed of different lipid mixtures patterned as diffraction gratings; (**d**) Clustering in principal component space of sensor responses to acetone, ethanol, and water demonstrate specific detection of the different organic vapors. The first two principal components account for 96.36% of the total variance in the set of solvent experiments.

As time progresses, each unique lipid grating reacts differently to vapor exposure. Figure 4a–c show the specific time progression of the different lipid formulations exposed to acetone, ethanol and water vapors, respectively. Once each lipid response was recorded, normalized diffraction intensities were extracted for each lipid formulation. We found that diffraction intensities of each lipid formulation at two times (20 and 40 s after vapor administration) resulting in 12 parameters representing the system’s fingerprint-provided good results. The test was repeated five times for three different solvents. These data were then transformed into principal component space and reduced to the first two principal components. It was found that the first two principal components together accounted for 96.36% of the data variation. The results of the principal component analysis are shown in Figure 4d. Clustering for each of the solvents demonstrates the specificity capabilities of the optical lipid gratings.

### 3.4. pH Detection

**Figure 5:**
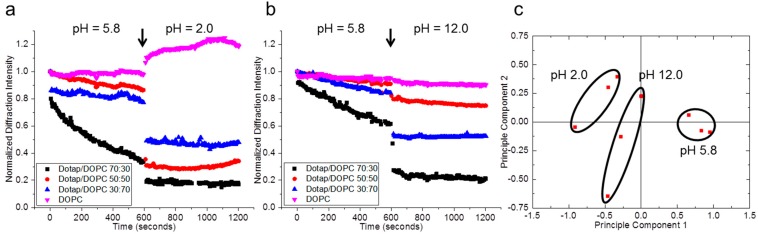
Detection of pH changes under water. Principal component analysis (PCA) results in separated clusters of lipid grating responses to different pH. (**a**,**b**) Normalized diffraction intensity of lipid multilayer gratings consisting of different charge. Upon immersion into deionized water of pH 5.8, the lipid gratings undergo different degrees of spreading to account for decreases in diffraction signal. Upon altering the pH after 10 min of incubation to either more acidic Figure 5a or basic Figure 5b conditions, changes in diffraction signal are realized after allowing adjusted solution to incubate for ten more minutes; (**c**) Clustering in principal component space suggests the lipid multilayer grating array chip can distinguish acidic and basic conditions. The first two principal components account for 86.46% of the total variance in the set of pH experiments.

In order to test the suitability for the grating arrays to detect analytes in aqueous solution, lipid gratings were first immersed in Millipore water with pH 5.8 for ten minutes before exposure to pH adjustments (Figure 5). At the 10 minute mark (denoted by an arrow in Figure 5a,b), the pH of the solution was adjusted to 2.0 (Figure 5a) or 12.0 (Figure 5b), or left at 5.8 depending on the trial. Tuning the pH to more acidic or basic conditions generally causes the lipid gratings to decrease their diffraction intensity with the exception of low pH (2.0) initially causing DOPC gratings to increase, before decreasing (b, Supplementary video 2). It is suggested here that lower pH causes protonation of the zwitterion DOPC, which may cause a charge induced swelling of the lipid gratings, while remaining physisorbed to the substrate. The gratings were left in more acidic or basic conditions for 10 min to complete the experiment. PCA was done in the same manner used for vapor detection. For 10 min incubations of pH 5.8, two normalized diffraction intensities were taken for each grating (100 s and 350 s). After the pH was adjusted to either 2.0 or 12.0, two more normalized diffraction intensities were taken (the normalization is with respect to the pH 5.8) again at 100 and 350 s. Based on the principal component analysis, separation was observed between the three different pHs, demonstrating the capability of lipid multilayer sensor arrays to be applicable to detecting concentration of hydrogen ions in solution. Two principal components account for 86.46% of the total variance in the set of pH experiments.

## 4. Conclusions/Outlook

Uniform patterning of nanoscale lipid gratings enable efficient, optical diffraction monitoring of lipid multilayers in situ. Based on the lipid grating sensor design, the grating itself acts as the transducing element, enabling real time detection of nanoscale changes in lipid multilayer topography. The lipid multilayer grating sensor chip demonstrated utility in detecting analytes in air by providing unique responses to different solvents and in solution by distinguishing environments composed of different quantities of hydrogen ions. It is anticipated that the cost-effective sensor array will be further established to include application for the specific detection of analytes and characterization of the binding.

## Supplementary Files

Supplementary File 1
